# Enhanced production of gamma-aminobutyrate (GABA) in recombinant *Corynebacterium glutamicum* by expressing glutamate decarboxylase active in expanded pH range

**DOI:** 10.1186/s12934-015-0205-9

**Published:** 2015-02-15

**Authors:** Jae Woong Choi, Sung Sun Yim, Seung Hwan Lee, Taek Jin Kang, Si Jae Park, Ki Jun Jeong

**Affiliations:** Department of Chemical and Biomolecular Engineering (BK21 Plus Program), KAIST, 335 Gwahagno, Yuseong-gu, Daejeon 305-701 Republic of Korea; Department of Biotechnology and Bioengineering, Chonnam National University, 77 Yongbong-ro, Buk-gu, Gwangju 500-757 Republic of Korea; Department of Chemical and Biochemical Engineering, Dongguk University-Seoul, 30 Pildong-ro 1-gil, Jung-gu, Seoul 100-715 Republic of Korea; Department of Environmental Engineering and Energy, Myongji University, 116 Myongji-ro, Cheoin-gu, Yongin, Gyeonggido 449-728 Republic of Korea; Institute for the BioCentury, KAIST, 335 Gwahagno, Yuseong-gu, Daejeon 305-701 Republic of Korea

**Keywords:** *Corynebacterium glutamicum*, Gamma-aminobutyrate, Glutamate, Glutamate decarboxylase, Biotin, Fed-batch cultivation

## Abstract

**Background:**

Gamma-aminobutylate (GABA) is an important chemical in pharmacetucal field and chemical industry. GABA has mostly been produced in lactic acid bacteria by adding *L*-glutamate to the culture medium since *L*-glutamate can be converted into GABA by inherent *L*-glutamate decarboxylase. Recently, GABA has gained much attention for the application as a major building block for the synthesis of 2-pyrrolidone and biodegradable polyamide nylon 4, which opens its application area in the industrial biotechnology. Therefore, *Corynebacterium glutamicum*, the major *L*-glutamate producing microorganism, has been engineered to achieve direct fermentative production of GABA from glucose, but their productivity was rather low.

**Results:**

Recombinant *C. glutamicum* strains were developed for enhanced production of GABA from glucose by expressing *Escherichia coli* glutamate decarboxylase (GAD) mutant, which is active in expanded pH range. Synthetic P_H36_, P_I16_, and P_L26_ promoters, which have different promoter strengths in *C. glutamicum*, were examined for the expression of *E. coli* GAD mutant. *C. glutamicum* expressing *E. coli* GAD mutant under the strong P_H36_ promoter could produce GABA to the concentration of 5.89 ± 0.35 g/L in GP1 medium at pH 7.0, which is 17-fold higher than that obtained by *C. glutamicum* expressing wild-type *E. coli* GAD in the same condition (0.34 ± 0.26 g/L). Fed-bath culture of *C. glutamicum* expressing *E. coli* GAD mutant in GP1 medium containing 50 μg/L of biotin at pH 6, culture condition of which was optimized in flask cultures, resulted in the highest GABA concentration of 38.6 ± 0.85 g/L with the productivity of 0.536 g/L/h.

**Conclusion:**

Recombinant *C. glutamicum* strains developed in this study should be useful for the direct fermentative production of GABA from glucose, which allows us to achieve enhanced production of GABA suitable for its application area in the industrial biotechnology.

## Background

Gamma-aminobutyrate (GABA), is a non-protein amino acid, which is synthesized in many microorganisms, plants and animals [1]. GABA has been used as a bioactive component in various foods and pharmaceutical products due to its potential in controlling neurotransmitter signal and lowering blood pressure in human [2-4]. In addition, GABA has been used as a major building block for the synthesis of 2-pyrrolidone and biodegradable polyamide nylon 4, which opens its application area in the industrial biotechnology [5,6]. Thus, the development of strategy for economical production of GABA become an important issue to meet its expanding demand in the various areas from a high value added business such as pharmaceuticals to biomass-based industry such as bioplastics and biochemicals.

Since GABA can be produced by decarboxylation of glutamate, the strategy of biological production of GABA has been based on the efficient conversion of glutamate into GABA by glutamate decarboxylase (GAD; EC 4.1.1.15). Purified GAD and natural and recombinant microorganisms expressing GAD have been employed for the conversion of glutamate monosodium salt (MSG) or glutamic acid to GABA. Recent studies have suggested that GABA can be efficiently produced from L-glutamate by some lactic acid bacteria (LAB) [7,8] and recombinant *Escherichia coli* [5,9,10]. Basically, it is needed to develop additional enzymatic and whole cell mediated conversion of MSG into GABA when fermentation-derived MSG is used for the production of GABA. Even though GABA can be efficiently produced from MSG by enzymatic and whole cell biocatalytic processes, these processes might be complex to design for industrial-scale production of GABA compared with direct fermentative production of GABA from glucose due to the need for additional processes to link MSG and GABA production process. Recently, *C. glutamicum* has drawn much attention as an alternative host for direct fermentative production of GABA from renewable carbon sources since it has already been used as an industrial host strain for the production of amino acids such as MSG and Lysine by fermentation with an annual production of more than 1.5 million tons [11-14]. *C. glutamicum* is a Gram (+), non-pathogenic and non-spore forming bacterium that has been traditionally used for the production of various amino acids [15-18] and is generally regarded as safe (GRAS) which is suitable for the production of foods and pharmaceutical products [19]. Recently, fermentative production of GABA from renewable carbon sources has been reported by recombinant *C. glutamicum* strains. Recombinant *C. glutamicum* expressing *Lactobacillus brevis* glutamate decarboxylase successfully produced GABA up to 26 g/L in 120 h fermentation [12]. Also, *C. glutamicum* expressing *E. coli* glutamate decarboxylase, which was further engineered by the deletion of the *pknG* gene encoding serine/threonine protein kinase G, resulted in the production of 31 g/L of GABA in 120 h fermentation [11]. In these studies, wild-type glutamate decarboxylases that have optimal pH around 4.5 for their decarboxylation of glutamate were employed for the production of GABA, but the optimal culture pH of *C. glutamicum* strain for its growth was much higher than 4.5. Due to the discrepancy of optimal pH between activity of GAD and cell growth, GABA could not be produced during the exponential growth phase in which both the cell growth and the synthesis of glutamate, the direct substrate for GABA, were active. The direct conversion for GABA was initiated in stationary growth phase when the culture pH was relatively lower than initial pH [14]. GABA was produced and this was the main reason for relatively long time cultivation over 120 h, which resulted in low GABA productivity.

In this study, we have developed recombinant *C. glutamicum* strains that efficiently produce GABA in culture pH around 5 ~ 7 by employing *E. coli* GAD mutant active in expanded pH range, which can ultimately provide balanced condition for cell growth and GABA production. Optimal expression conditions of *E. coli* GAD mutant (Glu89Gln/Δ452-466), in which C-terminal region is removed and contains mutation of Glu89Gln [20], were designed based on synthetic promoters active in recombinant *C. glutamicum* [21]. Finally, fed-batch cultivation strategies of recombinant *C. glutamicum* expressing *E. coli* GAD mutant were developed for enhanced production of GABA from glucose.

## Results

### Comparison of activity of *E. coli* wild type and mutant GADs in recombinant *C. glutamicum*

**Figure 1:**
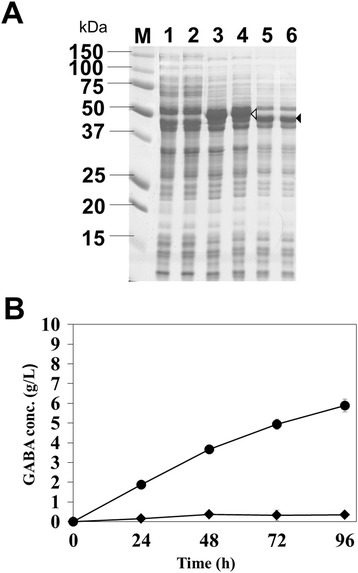
**Comparison of GABA production in** ***C. glutamicum*** **producing wild type GAD or mutant GAD in flaks cultivation. (A)** SDS-PAGE analysis of production of wild type GAD and mutant GAD. Lane M, protein size markers (kDa). Lanes 1 and 2, *C. glutamicum* harboring pCES208. Lanes 3 and 4, *C. glutamicum* harboring pHGwt. Lanes 5 and 6, *C. glutamicum* harboring pHGmut. Lanes 1, 3, and 5, total protein fractions. Lanes 2, 4, and 6, soluble protein fractions. The open and closed arrowheads indicate the band of wild type GAD and mutant GAD, respectively. **(B)** Time profiles of GABA concentration. Diamond and circle symbols indicate *C. glutamicum* harboring pHGwt and pHGmut, respectively.

For biosynthesis of GABA, GAD plays the key role but its low optimal pH range (~ pH 4) has been critical limitation because its optimal pH for catalytic activity is not suitable for the growth of most recombinant hosts including *E. coli* and *C. glutamicum* [22]. Recently, *E. coli* GadB mutant (Glu89Gln/Δ452-466) having catalytic activity in broadened pH range up to pH 7 was developed by engineering *E. coli* GadB [20], which might allow us to achieve enhanced production of GABA since culture conditions for cell growth and synthesis of L-glutamate, the direct precursor of GABA, can be optimized for GABA production in broadened culture pH conditions. First, we constructed recombinant *C. glutamicum* strains expressing *E. coli* wild-type GadB and engineered *E. coli* GadB under the strong synthetic H36 promoter and compared these strains in the respective of GABA production in GP1 medium containing 500 μg/L biotin at 30°C and initial pH 7. Under the strong and constitutive P_H36_ promoter, both the *gadB* mutant gene and the wild type *gadB* gene were expressed well and GAD proteins could be produced with high solubility (Figure 1A). Recombinant *C. glutamicum* harboring pHGmut successfully produced GABA to the concentration of 5.89 ± 0.35 g/L, but recombinant *C. glutamicum* harboring pHGwt produced much lower GABA to the concentration of 0.34 ± 0.26 g/L (Figure 1B). It is well-known that *C. glutamicum* expressing wild type *gadB* genes could produce GABA when they were cultivated at low pH conditions [13,14]. But, in the near-neutral pH condition, the wild type GAD has much lower activity, and as expected, the small amount of GABA was produced by the expression of wild type GAD in *C. glutamicum* (Figure 1B).

### Effect of promoter strength on GABA production in recombinant *C. glutamicum*

**Figure 2:**
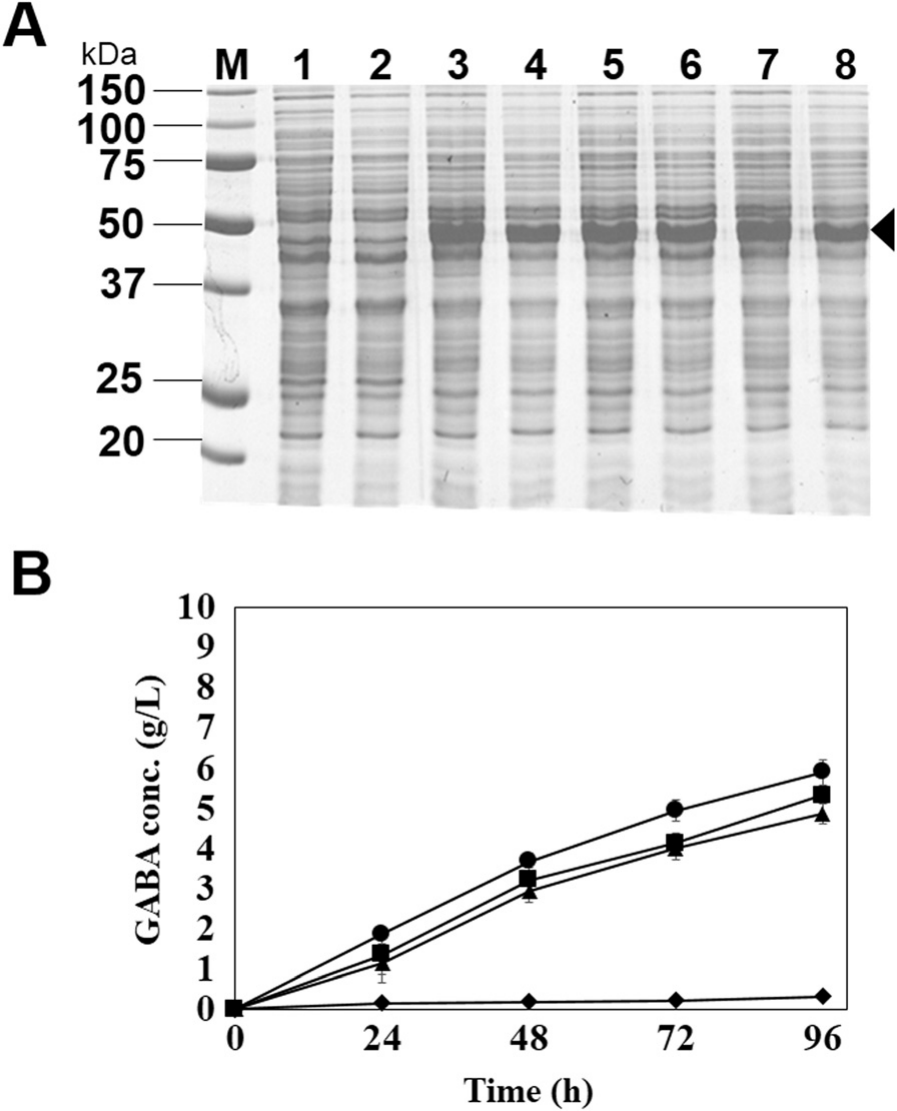
**Comparison of three different promoters for gene expression and GABA production in** ***C. glutamicum*** **. (A)** SDS-PAGE analysis of mutant GAD gene expression under the three synthetic promoters (P_L26_, P_I16_, or P_H36_). Lane M, protein size markers (kDa). Lanes 1 and 2, *C. glutamicum* harboring pCES208.Lanes 3 and 4, *C. glutamicum* harboring pLGmut. Lanes 5 and 6, *C. glutamicum* harboring pIGmut. Lanes 7 and 8, *C. glutamicum* harboring pHGmut. Lanes 1, 3, 5, and 7, total protein fractions. Lanes 2, 4, 6, and 8, soluble protein fractions. **(B)** Time course of GABA concentration during flask cultivation of *C. glutamicum*. Triangle, square, circle and diamond symbols indicate *C. glutamicum* harboring pLGmut, pIGmut, pHGmut and pHGwt respectively. Each point represents the average of three independent experiments.

To achieve the high-level production of biomolecules in bacterial host, it is one of the important issues to construct the gene expression system which expresses the enzymes responsible for the production of target products with high solubility and functionality. In general, the use of strong promoter is preferred for overproduction of such enzymes, but in some cases, the overproduction of enzymes caused the insoluble aggregates which do not have any biological functions. Therefore, it is needed to find the proper promoter which allows the optimal gene expression and high-level production of target molecules, and for this purpose, we have examined promoters which have different promoter strengths. Previously, we succeeded in the isolation of 20 synthetic promoters which can mediate the constitutive gene expression in recombinant *C. glutamicum* [21]. Among 20 synthetic promoters, two more promoters, P_L26_ and P_I16_, were chosen for further examination since they have different promoter strengths in the order of P_L26_, P_I16_, and P_H36_ [21]. Each construct (pLGmut, pIGmut, or pHGmut) was transformed into *C. glutamicum* and, gene expression and GABA production were examined in flask cultivation (30°C and pH 7). *E. coli gadB* mutant gene was well expressed under all promoters with high solubility (Figure 2A). But the expression level under each promoter was very similar and so it was not easy to choose the promoter for gene expression. For further evaluation of three synthetic promoters, the culture supernatants were collected periodically during the cultivation, and the GABA production yields were analyzed. During cultivations, the culture supernatants were collected periodically and the GABA production yields were analyzed. All the recombinant *C. glutamicum* harboring pLGmut, pIGmut, and pHGmut, respectively, successfully produced GABA and GABA concentration continued to increase during cultivation. It was also found that GABA concentrations obtained by each recombinant *C. glutamicum* were highly correlated with the strength of promoters although gene expression levels under three promoters were similar. Among three promoters, the use of strong promoter (P_H36_) resulted in the highest GABA concentration (5.89 ± 0.35 g/L) than those of other promoters (5.32 ± 0.04 g/L under P_I16_ and 4.87 ± 0.15 g/L under P_L26_) after 96 h (Figure 2B). Therefore, in all following works, the strong synthetic promoter (P_H36_) was used for the expression of *gadB* mutant gene in *C. glutamicum*.

### Effect of biotin concentration on GABA production

**Figure 3:**
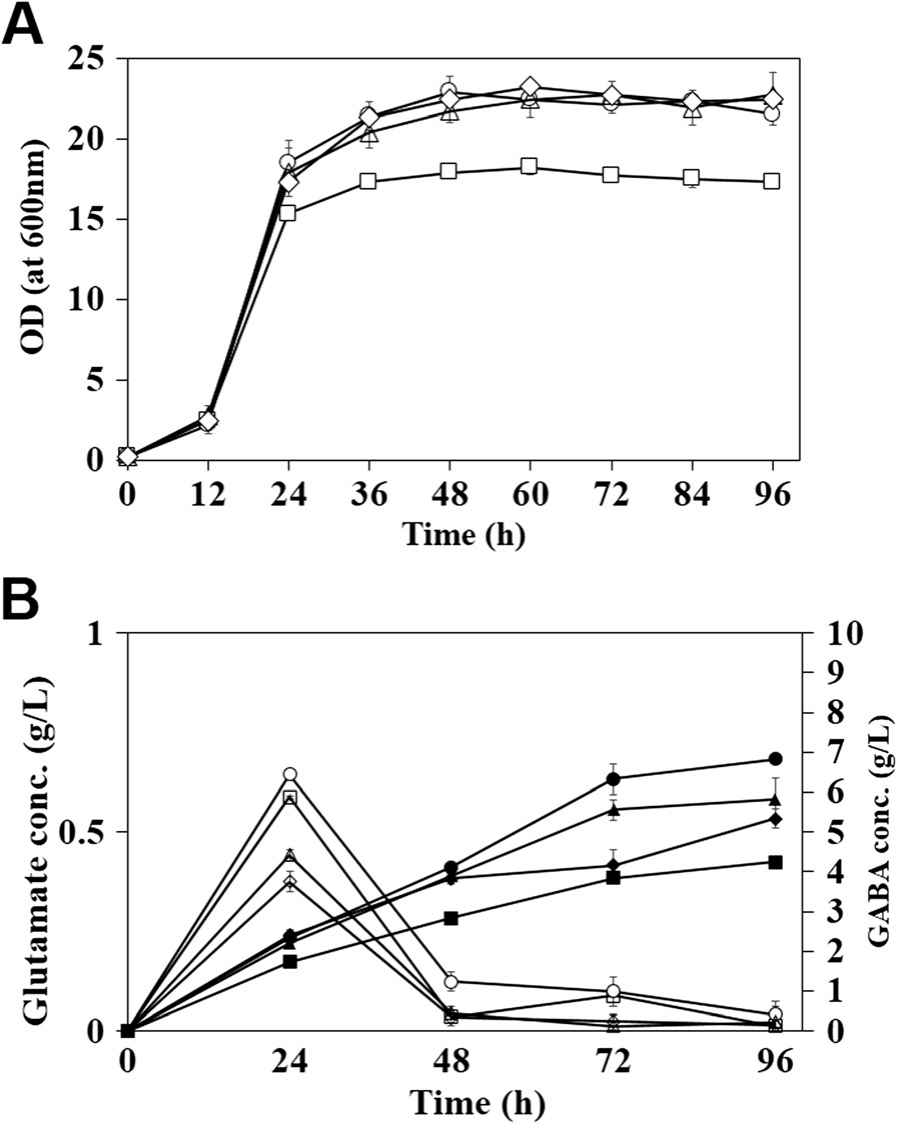
**Time profiles of cell density and GABA concentrations during flask cultivations with different biotin concentrations.** Square, circle, triangle and diamond symbols indicate 1 μg/L, 50 μg/L, 100 μg/L, and 500 μg/L, respectively. **(A)** Time profiles of cell growth at four different biotin concentration conditions. **(B)** Open and closed symbols indicate the L-glutamate concentrations and GABA concentrations, respectively. Each point represents the average of three independent experiments. Each point represents the average of three independent experiments.

For the enhanced production of GABA, the pool of its direct precursor, *L-*glutamate, should be increased in a production host [11]. It is well known that overproduction of *L*-glutamate is induced in *C. glutamicum* under the biotin-limited conditions and the low concentration of biotin in culture medium can give a positive effect on GABA production [23-26]. However, *C. glutamicum* is a biotin auxotrophic bacterium, which requires high-level concentration of biotin for cell growth, thus, it is necessary to optimize a biotin concentration for the enhanced production of GABA without cell growth retardation. To find the optimal concentration of biotin, *C. glutamicum* harboring pHGmut was cultivated in the GP1 medium containing four different biotin concentrations; 1 μg/L, 50 μg/L, 100 μg/L and 500 μg/L. In these experiments, the initial pH in the medium was adjusted to 7. In all concentrations of biotin except for 1 μg/L, cells exhibited similar growth patterns and could grow up to 20~22 of OD_600_ (Figure 3). In contrast, supplementation of the 1 μg/L of biotin resulted in the retardation of cell growth and lower final cell density (17 of OD_600_). The highest GABA concentration of 6.32 ± 0.38 g/L was obtained by the cultivation of recombinant *C. glutamicum* (pHGmut) in GP1 medium supplemented with 50 μg/L of biotin (Figure 3). In each cultivation, the level of glutamate was also determined and we found that the supplementation of 50 μg/L biotin exhibited the highest level of glutamate in the early stage (24 hr) and most of them was consumed in the period of GABA production (Figure 3B). This result clearly indicates that the optimization of biotin concentration can be one of the critical factors in the production of GABA by *C. glutamicum*.

### Effect of pH on GABA production

**Figure 4:**
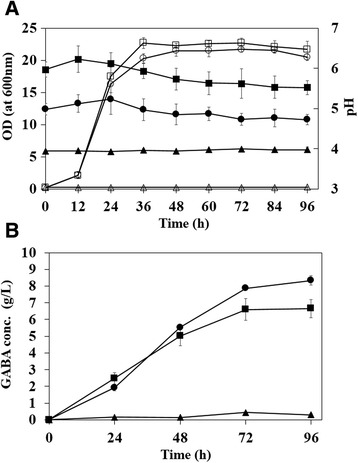
**Effect of pH on cell growth and GABA production in flask cultivation of** ***C. glutamicum*** **. (A)** Time profiles of cell growth and pH change at four different pH conditions. Triangle, circle and square symbols indicate the pH 4, pH 5 and pH 6, respectively. The open and closed symbols indicate the cell density and pH change, respectively. **(B)** Time profiles of GABA concentrations during cultivations. Triangle, and circle and square symbols indicate pH 4, pH 5 and pH 6, respectively. Each point represents the average of three independent experiments.

In contrast to wild type GAD having optimal pH around 4 [20], the pH spectrum for catalytic activity of the mutant GAD was expanded to near-neutral pH range, which is also optimal for cell growth [20]. Even though mutant GAD has catalytic activity in the range of pH 4 and pH 7, it was reported that specific activity of mutant GAD was gradually decreased to almost zero as the reaction pH increased from 4 to 8 [20]. Also, synthesis of glutamate, the precursor of GABA, might be different depending on the culture pH for the cultivation of recombinant *C. glutamicum*. Thus, cultivations of recombinant *C. glutamicum* harboring pHGmut were examined at three different pH conditions (pH 4, pH 5 and pH 6) to find the optimal pH for GABA production, and the results of cell growth and GABA production were compared with those of cultivation at pH 7. In cultivation at pH 4, cells did not show any growth (Figure 4A) and GABA concentration was also very low (0.29 ± 0.04 g/L) (Figure 4B). In contrast, cultivation at higher pH conditions (pH 5 and pH 6) resulted in much better cell growth than that of cultivation at pH 4 (Figure 4B), but a little slower growth than that of cultivation at pH 7 (Figure 4A). At both pH conditions, cells grew well up to 19 and 21 of OD_600_, respectively. During the cultivation at pH 5, the pH was well maintained in entire cultivation for 96 h, but, cultivation at pH 6 showed a little decrease of pH to 5.5 in late stage of cultivation (Figure 4A). At both pH conditions, the concentrations of GABA produced during cultivations gradually increased (Figure 4B). Particularly, cultivation at pH 5 resulted in higher GABA concentration of 8.34 ± 0.26 g/L than those obtained in the cultivations at higher pH conditions (pH 6 and pH 7). In cultivation at pH 6, a little higher GABA concentration of 6.60 ± 0.64 g/L was obtained than that at pH 7. Also, volumetric productivity of 0.11 g/L/h obtained in cultivation at pH 5 was higher than those obtained at pH 6 (0.09 g/L/h) and pH 7 (0.08 g/L/h).

### Fed-batch cultivations for GABA production

**Figure 5:**
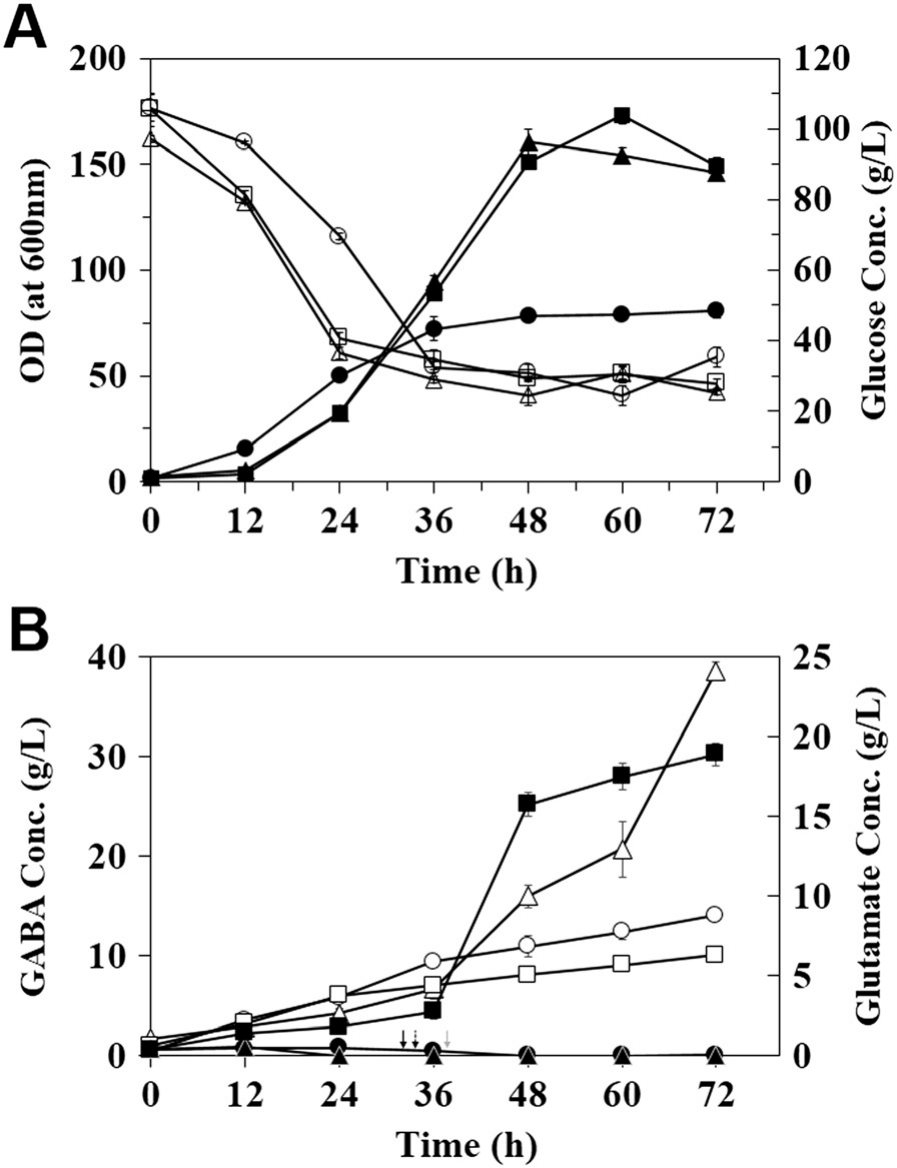
**Fed-batch cultivation for GABA production with different pH conditions. (A)** Time profiles of cell growth and glucose concentration. Circle, triangle, and square symbols indicate pH 5, pH 6 and pH 7, respectively. Open and closed symbols indicate the glucose concentration and cell density, respectively. **(B)** Time profiles of L-glutamate and GABA concentrations. Circle, triangle, and square symbols indicate pH 5, pH 6 and pH 7, respectively. Open and closed symbols indicate the GABA and glutamate concentrations, respectively. The first time points for glucose feeding were indicated by gray-solid (pH 5), black-dashed (pH 6) and black-solid (pH 7) arrows.

Fed-batch cultivations of recombinant *C. glutamicum* harboring pHGmut were carried out for the high-level production of GABA from glucose in 5 L lab-scale bioreactor containing GP1 medium supplemented with 50 μg/L of biotin. In cultivation at pH 5, cells grew up to 76.6 of OD_600_ in 72 h and cell specific growth rate (*μ*) was 0.055 h^−1^ (Figure 5A). The initial glucose (100 g/L) was gradually consumed during cultivation and glucose concentration was maintained at 30 g/L by periodically supplying nutrient feeding solution (Figure 5A). It was found out that GABA was produced even in the exponential cell growth phase. GABA concentration reached 14.0 ± 0.46 g/L in 72 h and the maximum volumetric productivity was 0.194 g/L/h (Figure 5B). Total amount of glucose supplied was 372 ± 2.9 g and the molar yield of GABA from glucose was 0.197 mol/mol glucose. In the early growth phase, small amount of *L*-glutamate was accumulated but after entering to the mid-log growth phase (36 h), the concentration of *L*-glutamate was maintained at almost zero (Figure 5B).

Fed-batch cultivation of *C. glutamicum* harboring pHGmut was also performed at pH 6 condition. At pH 6, cells exhibited much faster cell growth than that at pH 5. During exponential growth phase, cell specific growth rate (*μ*) was 0.11 h^−1^ which was almost 2-fold faster than that at pH 5 (Figure 5A). The maximum cell density of 167 of OD_600_ was also much higher than that at pH 5. As cell entered to exponential growth phase (in 24 h), the glutamate concentration in culture medium was maintained at almost zero (Figure 5B). As cell grew exponentially, GABA production also gradually increased. GABA concentration of 38.6 ± 0.85 g/L was obtained in 72 h culture, which was 2.7-fold higher than that at pH 5 and the maximum volumetric productivity was 0.536 g/L/h (Figure 5B). Total amount of glucose supplied was 482 ± 3.4 g and the molar yield from glucose was 0.560 mol/mol glucose.

Finally, fed-batch cultivation of *C. glutamicum* for GABA production was performed in pH 7. At pH 7, a specific growth rate (*μ* = 0.10 h^−1^) similar to that at pH 6 was obtained the maximum cell density reached to 162 of OD_600_ in 60 h, which were similar to those at pH 6 (Figure 5A). While the glutamate concentration was maintained at almost zero during fed-batch cultivation at pH 5 and 6, it was found out that significant amount of *L*-glutamate was accumulated during fed-batch culture at pH 7. Accumulation of glutamate was detected after 36 h cultivation, and its concentration reached 18.9 ± 0.71 g/L at the end of cu-ltivation (Figure 5B). According to accumulation of glutamate, relatively low GABA concentration of 9.86 ± 0.52 g/L was obtained and maximum volumetric productivity was 0.136 g/L/h (Figure 5B). Total amount of glucose supplied was consumed 498 ± 7.4 g and the molar yield from glucose was 0.103 mol/mol glucose.

## Discussion

For the enhanced production of target products such as recombinant proteins, biochemical and biopolymers, the efficient expression system of inherent and heterologous genes needs to be constructed. For this purpose, the choice of proper promoter is an important issue but, unfortunately, only a few promoters are available in *C. glutamicum* and it is not easy to find the proper promoter for high-level gene expression in *C. glutamicum* [27,28]. Previously, we succeeded in the isolation of fully synthetic promoters which made constitutive gene expression with different strengths in *C. glutamicum* [21,29]. By examining different promoters, the best system for the high-level expression of target gene in *C. glutamicum* could be constructed [21]. In this work, we examined three synthetic promoters having different promoter strength, which have been screened in previous study [21] for the construction of recombinant *C. glutamicum* to achieve enhanced production of GABA from glucose. Among three synthetic promoters, P_L26_, P_I16_, and P_H36_, which have different promoter strengths in the order of P_L26_, P_I16_, and P_H36_, the use of P_H36_ promoter allowed the high-level expression of GAD along with much enhanced production of GABA. This can be another example to prove the usefulness of the synthetic promoters for high-level gene expression in *C. glutamicum*.

For the higher production of target products including recombinant proteins and metabolites, cells need to be cultivated to high cell density (generally higher than OD = 100), and for this purpose, fed-batch cultivations have widely been used in which pH was set at optimal pH for cell growth. However, instead of fed-batch cultivations, batch cultivations have been used for GABA production, in which pH was not adjusted during the cultivation for the production of GABA due to the characteristics of wild-type GAD, the main catalyst for the conversion of glutamate into GABA. Since the wild-type GAD has optimal catalytic activity only in low pH range around 4 and it has almost no enzyme activity at pH higher than 4, thus, two stage cultivation strategy has generally been employed for GABA production to satisfy the conditions for cell growth and GABA production. The batch cultivations were divided to two stages: i) cell growth stage and ii) GABA production stage. When the culture pH was in the range suitable for cell growth, cells grew well without much GABA production since GAD has very little catalytic activity. As the cells entered into the stationary phase, in which pH began to decrease without further cell growth, GABA production began to increase since GAD became active in the low pH condition. However, high-level production of GABA could not be achieved in such batch type cultivations since high cell density of cells expressing GAD gene could not be achieved and GABA was produced only in stationary growth stage in which GABA production efficiency was not high. Therefore, the quantitative amount of GABA of around 27 g/L was only produced in the cultivation operated for long time over 100 h [12,20,30].

In this study, for the enhanced production of GABA in *C. glutamicum*, we employed *E. coli* GAD mutant that has catalytic activity in expanded pH range up to pH 7. In the flask cultivation at pH 7, the use of GAD mutant exhibited more than 17-fold higher production of GABA than that of wild-type GAD (Figure 1). With GAD mutant, we also examined four different pH conditions (pH 4, 5, 6 and 7) to find the optimal pH condition, which might allow us to design for the conditions to support higher cell growth as well as higher production of GABA. By the cultivation of recombinant *C. glutamicum* at culture pH of 4, very little amount of GABA was produced due to the poor cell growth even though GAD is reported to be highly active at pH 4. As the culture pH increased to 5, 6, and 7, cell growth rates and final cell density were not much different but, a little higher GABA concentration was obtained by cultivation at pH 5 (Figure 4 B). In flaks cultivation, we set only the initial pH in the medium and the pHs were not adjusted during the cultivation. It is known that the production of GABA resulted in the increase of pH due to the consuming protons in a decarboxylation reaction [31]. But, pH in the media can be also affected by other factors including media compositions and formation of organic acids byproducts (acetate, lactate, citrate etc.) which cause the decrease of pH in the media [32]. Actually, the initial pH in the medium decreased gradually during the cultivation, and the decreased pH might give an inhibitory effect on the cell growth. So, in flask cultivations, there were not big difference in cell density and so, under the similar cell density, more GABA might be produced at lower pH condition because the engineered GAD also has higher activity at lower pH than higher pH although it has broad pH range for catalytic activity. However, in the fed-batch cultivation, pH could be adjusted to set-point during the entire cultivation unlike as flask cultivations. Under each pH condition, cells exhibited much different cell growth profiles and, as expected, much higher growth rate and cell density (up to OD_600_ of 160) could be obtained at higher pHs (pH 6 and 7) than pH 5 (OD_600_ of 72). With this higher cell density, much higher GABA production could be also obtained although the GAD has lower activity at pH 6 than pH 5. It was also clearly observed that the cell growth and GABA production profiles were well associated at both pH conditions (pH 5 and pH 6). During the exponential growth phase, GABA production could initiate and GABA concentration was gradually increased by the constitutive expression of the engineered GAD gene under the strong synthetic promoter (P_H36_) (Figure 5B). Thus, high GABA concentration of 38.6 ± 0.85 g/L could be obtained by 72 h cultivation, which was much shorter than 96~168 h cultivation times in previous reports [11,12]. Also, the high productivity of 0.536 g/L/h could be obtained and, to the best of our knowledge, this productivity is the highest record in GABA production.

For the efficient production of target products, the supply and consumption rates of precursors for the target products should be balanced enough to support high-level production of target products without much accumulation of unused precursors. In this study, the optimal culture pH for the production of glutamate, the direct precursor of GABA and the conversion of glutamate into GABA might be different. The former is proportional to culture pH up to 7 and the latter is inversely proportional to culture pH. Also, the optimal condition for cell growth is another factor to consider for enhanced production of GABA. It has been reported that optimal pH of *C. glutamicum* growth is pH 7 to 8.5 and inhibition of TCA occurred at low pH [33]. Since glutamate is mainly converted from 2-oxoglutarate in TCA cycle, optimal culture pH of glutamate production can be found in the point that TCA cycle is inhibited without much cell growth retardation to supply 2-oxoglutarate for glutamate synthesis [34]. Besides efficient supply of glutamate as precursor of GABA, optimal culture condition for the conversion of glutamate into GABA by mutant GAD should be designed for enhanced production of GABA during cultivation of recombinant *C. glutamicum*. As shown in the fermentation profiles obtained at the culture pH 5, 6, and 7, the accumulation of glutamate occurred in all the fed-bath cultivation, the extents of which were different in each cultivation. In pH 5 and 6, glutamate accumulation was only observed in the early stage of cultivation and glutamate was rapidly consumed for the production of GABA resulting in no further accumulation of glutamate in active GABA synthesis phase. However, during the entire cultivation of recombinant *C. glutamicum* at pH 7, large amount of glutamate was accumulated in the culture medium up to 18.9 ± 0.71 g/L, which resulted in rather low GABA concentration of 9.86 ± 0.52 g/L. These fermentation results clearly suggested several important factors affecting the production GABA through glutamate from glucose should be considered to design the optimal culture condition for enhanced production of GABA, which include cell growth, synthesis of glutamate and rapid conversion of synthesized glutamate into GABA without much accumulation. In the cultivation at pH 6~7, cells grew better than that at pH 5, and much more glutamate could be produced (particularly in the stationary growth phase). In the cultivation at pH 6, mutant GAD was more active than at pH 7 and so, most glutamate produced in stationary phase could be converted to GABA which might be also one reason for higher production of GABA during the stationary growth phase although mutant GAD was already produced and GABA began to be produced in the exponential growth phase (Figure 5B). In the cultivation of recombinant *C. glutamicum* at pH 7, rather low activity of mutant GAD that is not enough to catch up with the glutamate synthesis seems to be main reason for low production of GABA along with much accumulation of unused glutamate in the culture medium. In the fed-batch cultivation at pH 5 which is not preferable condition for cell growth, much less glutamate might be synthesized, which resulted in much lower production of GABA compared with that of pH 6. In the culture pH 6, increased GAD activity could be balanced for glutamate synthesis to result in high-level production of GABA up to 38.6 ± 0.85 g/L.

## Conclusions

In this study, we have developed recombinant *C. glutamicum* strains for the enhanced production of GABA by employing mutant GAD that has catalytic activity in broadened pH range, which allowed us to obtain growth associated production of GABA by modulating cell growth, glutamate synthesis and conversion of glutamate into GABA in balanced level. These were achieved by strong synthetic promoter system supporting stable expression of the engineered *gadB* gene in high-level in recombinant *C. glutamicum* and optimization of culture conditions to support both high cell growth and high GABA production such as culture pH and biotin concentration. To our best knowledge, the productivity of 0.536 g/L/h for GABA synthesis is the highest among those obtained in recombinant *C. glutamcium* from glucose. Recently, several important nylon monomer precursors such as 5-aminovalerate, cadaverine, and putrescine have been produced by recombinant *E. coli* and *C. glutamicum* strains employing different cultivation strategies using lysine and glutamate as precursors [5,35-41]. Since *C. glutamicum* is one of important industrial host strains for the production of amino acids, metabolic engineering strategies developed in this study can be further expanded for the development of recombinant *C. glutamicum* to produce such nylon monomer precursors from renewable resources.

## Materials and methods

### Bacterial strains, media and culture conditions

The bacterial strains and plasmids used in this study are listed in Table 1. *E. coli* XL1-Blue was used as the host for gene cloning and plasmid maintenance, and *C. glutamicum* ATCC 13032 was used as the host strain for the expression of GAD and for the production of GABA. *E. coli* was grown in Luria-Bertani (LB) medium containing 10 g/L tryptone, 5 g/L yeast extract and 5 g/L NaCl at 37°C. For cultivation of *C. glutamicum* in shake flasks, Brain Heart Infusion (BHI, BD) and GP1 medium were used. GP1 medium (pH 7.0) contained 50 g/L (NH_4_)_2_SO_4_, 1 g/L K_2_HPO_4_, 3 g/L urea, 0.4 g/L MgSO_4_·7H_2_O, 50 g/L peptone, 0.01 g/L FeSO_4_, 0.01 g/L MnSO_4_·5H_2_O, and 200 μg/L thiamine [14]. 0.1 mM Pyridoxal 5′-phosphate hydrate (PLP), biotin and glucose at different concentration were also added to GP1 medium to support the growth of recombinant *C. glutamicum*. Thiamine, biotin and PLP were prepared as stock solution using 0.22 μm filter membrane and were added before cell cultures. Glucose was also separately autoclaved and added to the concentration of 50 g/L in shake flask cultures. Kanamycin (Km, 25 μg/mL) was added to the culture medium.

**Table 1:** **Bacterial strains and plasmids used in this study**

**Strain**	**Relevant characteristics**	**Reference or source**
*E. coli* XL1-Blue	*recA1 endA1 gyrA96 thi-1 hsdR17 supE44 relA1 lac [F′ proABlacIq ZΔM15 Tn10 (Tet* ^*r*^ *)]*	Stratagene^a^
*E. coli* DH5α	*fhuA2 lac(del)U169 phoA glnV44 Φ80' lacZ(del)M15 gyrA96 recA1 relA1 endA1 thi-1 hsdR17*	Lab stock
*C. glutamicum*	Wild type	ATCC13032
**Plasmids**	**Relevant characteristics**	**Reference or source**
pET28b-GAD ΔCE89Q	*gadB* mutant (Glu89Gln/Δ452-466 gene)	[20]
pCES208	*E. coli-C. Glutamicum* shuttle vector, Km^r^	[40]
pCES-H36-GFP	pCES208 derivative, P_H36_, eGFP	[21]
pCES-I16-GFP	pCES208 derivative, P_I16_, eGFP	[21]
pCES-L26-GFP	pCES208 derivative, P_L26_, eGFP	[21]
pHGwt	pCES208 derivative, *gadB* (wt)	This study
pHGmut	pCES208 derivative, Glu89Gln/Δ452-466 gene	This study
pLGmut	pCES208 derivative, P_L26_, Glu89Gln/Δ452-466 gene	This study
pIGmut	pCES208 derivative, P_I16_, Glu89Gln/Δ452-466 gene	This study

^a^Stratagene, La Jolla, CA, USA.

### Construction of plasmids

Plasmids pCES-H36-GFP, pCES-I16-GFP, and pCES-L26-GFP, which were constructed using *E. coli*-*C. glutamicum* shuttle vector, pCES208 [42], have previously been described [21]. They contain synthetic strong H36, intermediate I16, and low L26 promoter, respectively, for the expression of target gene in desired level in recombinant *C. glutamicum* [21]. A polymerase chain reaction (PCR) was performed using the C1000™ Thermal Cycler (Bio-Rad, Hercules, CA, USA) with PrimeSTAR HS polymerase (Takara Bio Inc., Shiga, Japan). The nucleotide sequences of all the primers used in this study are listed in Table 2. The wild type *E. coli gadB* gene was amplified by PCR with primers Gad-del-C-F and Gad-wt–R from chromosomal DNA of *E. coli* DH5α. The *E. coli* mutant *gadB* gene for the expression of mutant GAD that contains one mutation of E89Q and C-terminal deletion from 452 bp to 466 bp was amplified by PCR with primers Gad-del-C-F and Gad-del-C–R from pET28b-GAD ΔCE89Q [20]. Both PCR products were digested with two restriction enzymes (*Bam*H1 and *Not*1) and then, cloned into the same restriction enzyme sites of pCES-H36-GFP which contains strong and constitutive promoter (P_H36_) to yield pHGwt and pHGmut, respectively. For the gene expression under the intermediate strength promoter (P_I16_), P_I16_ promoter was amplified from pCES-I16-GFP by PCR with primers I16-F and I16-R. The PCR product was digested with *Kpn*I and *Bam*HI, and then cloned into pHGmut to yield pIGmut. For the gene expression under the low strength promoter (P_L26_), P_L26_ promoter was amplified from pCES-L26-GFP by PCR with primers L26-F and L26-R. After digestion with *Kpn*I and *Bam*HI, PCR product was cloned into pHGmut to yield pLGmut. All plasmids were constructed in *E. coli* XL1-Blue and then transformed into *C. glutamicum* ATCC 13032 by electroporation using a Gene Pulser (Bio-Rad). Manipulation of DNA such as restriction enzyme digestion, ligation, and agarose gel electrophoresis were carried out using standard procedures [43].

**Table 2:** **List of primers used in PCR experiments** ^**a**^

**Primer name**	**Primer sequence (5′ to 3′)**
Gad-del-C-F	tgacga**ggatcc**atgggcagcagcc
Gad-del-C-R	ctgcta**tctaga**ttagtgatcgctgagatatttca
I16-F	tcgatc**ggtacc**agacaccgc
I16-R	gatcga**ggatcc**caataatatcctgtagc
L26-F	tcgatc**ggtacc**gtgagtttagagca
L26-R	gatcga**ggatcc**gatagtaatcctaacg
Gad-wt-R	tgcta**gcggccgc**ttaggtatgtttaaagctgttctgtt

^a^Restriction enzyme sites are shown in bold.

### Cultivation in shake flasks

Recombinant *C. glutamicum* strain was first inoculated in 10 mL brain heart infusion (BHI) medium containing Km (25 μg/mL) and was cultivated at 30°C for 24 h with 200 rpm. Then, 2 mL of pre-culture was transferred into 50 mL of GP1 medium supplemented with PLP, biotin, and Km in 250 mL shake flask for GABA production. Cells were cultivated at 30°C for 96 h with 200 rpm.

### Fed-batch cultivation

For seed culture, recombinant *C. glutamicum* harboring pHGmut was inoculated into 200 mL of GP1 medium containing 50 μg/L of biotin in 1 L baffled flask and was cultivated at 30°C for 48 h with 200 rpm. The seed culture was transferred into 2 L of fresh GP1 medium containing 100 g/L glucose in a 5-L jar bioreactor (BioCNS, Daejeon, Republic of Korea). During the cultivation, the temperature was maintained at 30°C. The pH and dissolved oxygen (DO) concentration were controlled at the set points by on-line monitoring. The DO concentration was maintained at 30% (*v/v*) by automatically increasing the agitation speed up to 1,200 rpm and then by mixing the pure oxygen through a gas mixer. Three different pHs (5.0, 6.0 and 7.0) were examined and pHs were controlled by adding 5 N ammonia solution. Glucose as a sole carbon source was fed by a peristaltic pump to maintain the concentration of glucose about 30 g/L during fed-batch culture. During the cultivation, cell growth was monitored by measuring the optical density at 600 nm (OD_600_) with a spectrophotometer (Optizen POP; Mecays, Deajeon, Republic of Korea).

### Protein preparation and analysis

After flask cultivation, cells were harvested by centrifugation at 6,000 rpm for 10 min at 4°C. The harvested cells were re-suspended in phosphate-buffered saline (PBS, 135 mM NaCl, 2.7 mM KCl, 4.3 mM Na_2_HPO_4_, 1.4 mM KH_2_PO_4_, pH 7.2) and were disrupted by sonication at 9 min at 50% pulse and 20% amplitude (Sonic, Vibra cell, USA). After centrifugation of cell lysates at 10,000 rpm for 10 min at 4°C, soluble proteins were collected from supernatant. In SDS-PAGE analysis, samples were loaded on 12% polyacrylamide gels. After the gel electrophoresis, the gels were stained with Coomassie brilliant blue [50% (*v/v*) methanol, 10% (*v/v*) acetic acid, 1 g/L Coomassie brilliant blue R-250] for 1 hr and destained using a destaining solution [10% (*v/v*) methanol, 10% (*v/v*) acetic acid].

### Analysis of cell growth, GABA, L-glutamate and glucose concentration

During the cultivation, the concentration of glucose in the medium was determined by glucose analyzer (YSI 2700 SELECT™ Biochemistry Analyzer, YSI Life Science, Yellow Springs, OH, USA). For analyzing the concentrations of GABA and *L*-glutamate, an appropriate volume of culture medium was centrifuged at 13,000 rpm and 4°C for 10 min and, the supernatant was applied to reversed phase high-pressure liquid chromatography (HPLC, LC-20 AD, CTO-20A, SPD-20A; Shimadzu, Japan) equipped with Zorbax Eclipse amino acid analysis (AAA) column (4.6 × 150 mm 3.5-Micron; Agilent, USA). The separation of samples was attained using a binary non-linear gradient with mobile phase A (10 mM Na_2_HPO_4_ – 10 mM Na_2_B_4_O_7_, pH 8.2) and mobile phase B (acetonitrile:MeOH:H_2_O 45:45:10 by volume). Elution condition was as follows: equilibration (1.9 min, 100% A), gradient (16.2 min 0-57% B, 3.7 min 57-100% B), cleaning (4 min, 100% B). The column temperature was maintained at 40°C. Samples were detected at UV 338 nm. The standard curve for GABA and *L*-glutamate was determined using same procedure for 4 standard solutions [0.125, 0.25, 0.5 and 1 g/L of GABA and *L*-glutamate (Sigma, Missouri, USA)].

## Abbreviations

GABA: Gamma-aminobutyrate
GAD: Glutamate decarboxylase
PLP: Pyridoxal 5′-phosphate hydrate
